# Fine Flounder (*Paralichthys adspersus*) Microbiome Showed Important Differences between Wild and Reared Specimens

**DOI:** 10.3389/fmicb.2017.00271

**Published:** 2017-02-24

**Authors:** Carolina Ramírez, Jaime Romero

**Affiliations:** Unidad de Alimentos, Laboratorio de Biotecnología de los Alimentos, Instituto de Nutrición y Tecnología de los Alimentos, Universidad de ChileSantiago, Chile

**Keywords:** microbiota, next-generation sequencing (NGS), microbiome, flounder, *Paralichthys*

## Abstract

The intestinal microbiota is involved in a wide range of biological processes that benefit the host, including providing nutrition and modulating the immune system. Fine flounder (*Paralichthys adspersus*) is a flatfish of commercial interest that is native to the Chilean coast. The high value of this flatfish has prompted the development of stock enhancement and aquaculture activities. Knowledge of microbiota may help to improve the cultivation of this species; however, few comparative studies have evaluated the intestinal microbiota composition in farmed versus wild fishes. Intestinal contents from wild and aquaculture fish were collected, and DNA was extracted. Subsequently, the V3-region of 16S rRNA was PCR amplified and sequenced using the Ion Torrent platform. The comparison between wild and aquaculture specimens revealed important differences in the composition of the microbiota. The most abundant phylum in wild flounder was *Proteobacteria*, with an average relative abundance of 68.1 ± 15.4%; in contrast, in aquaculture flounder, this phylum had an average relative abundance of 30.8 ± 24.1%. Reciprocally, the most abundant phylum in flounder aquaculture was *Firmicutes*, averaging 61.2 ± 28.4%; in contrast, this phylum showed low abundance in wild flounder, in which it averaged 4.7 ± 4%. The phylum *Actinobacteria* showed greater abundance in wild flounder, ranging from 21.7 ± 18.8%, whereas, it averaged only 2.7 ± 3.8% in aquaculture fish. Specific taxa that were differentially distributed between wild and aquaculture flounder were identified using a statistical approach. At the genus level, a total of four genera were differentially represented between the two conditions. *Bacillus* and *Pseudomonas* were more highly represented in aquaculture flounder, whereas *Arthrobacter* and *Psychrobacter* were observed in wild flounder. Furthermore, in both cases, predicted functions (metabolic pathways) indicated that those microbiota might provide beneficial effects for the host, but wild flounder showed more noteworthy pathways (EPA/DHA, SCFA, biotin). Our results highlight the differences in the microbiota composition between wild and reared fish. Knowing the composition of the intestinal microbiota of *P. adspersus* is the first step toward exploring the proper management of this species, as well as toward the development of probiotics and functional foods based on their requirements.

## Introduction

Our current knowledge of gut microbiota composition and microbiota-host interactions in fish remains limited. Recent evidence has demonstrated that fish gut communities typically cluster with gut communities from mammals and insects (Sullam et al., 2012). Furthermore, these authors also identified a significant association between intestinal microbiota composition and fish taxonomy. This suggests potential co-evolution of fish and their gut microbiota, raising the possibility that fish were the first to evolve symbioses resembling those found in gut-fermenting mammals.

Meanwhile, aquaculture has become one of the fastest-growing animal production sectors in the world. Due to the increasing global population and limited fishery resources, aquaculture has increased its contribution to the per capita seafood consumption (44%, Fisheries and Aquaculture Topics, 2016). Currently, world aquaculture production is dominated by freshwater species, representing 56.4%; other reared fish provide a minor contribution (marine fishes represent only 3%). Thus, aquaculture activities are rapidly expanding worldwide and incorporating new species, and Latin American countries are starting to explore new species for aquaculture production. In flatfish aquaculture, Chile has started adapting technologies for the culture of turbot (*Psetta maxima*) and olive flounder or hirame (*Paralichthys olivaceus*) and has made a special effort to develop rearing technologies for endemic Chilean flounder or fine flounder (*Paralichthys adspersus*). This flatfish is a common inhabitant of gulfs and bays with sandy bottoms, and it has a wide geographical distribution from Paita, latitude 6° south, Perú, to Golfo de Arauco, latitude 36° south in Chile (Sielfeld et al., 2003).

One of the main challenges for diversification in aquaculture facilities is the control of diseases, particularly during early life stages. This is also an unresolved issue in current intensive aquaculture, resulting in partial or total loss of production. Furthermore, intensive aquaculture has shown severe problems associated with bacterial diseases, treatment of which has required the use of antimicrobials. In the case of *P. adspersus*, vibriosis is the most common bacterial disease in early life stages (Qiao et al., 2013). Several authors have emphasized the possible negative effects of intensive use of antimicrobial agents in aquaculture, increasing the risk of antibiotic resistance in pathogenic and environmental bacteria, which may be a major threat to the health of aquatic ecosystems and even human beings (Dang et al., 2009; Dang and Lovell, 2016). Finding alternatives to antibiotic treatments is an active research field, especially in aquaculture (Romero et al., 2012). In this context, the innate immune system of teleost fish plays a crucial role in fighting infectious diseases, and interest in basic research on this topic has increased in recent years (Montalban-Arques et al., 2015). Similar to mammals, fish also harbor gut microbiota, which may be a key aspect of the modulation of the host innate immune system (Llewellyn et al., 2014).

Despite the substantial roles that intestinal microbiota play in host metabolism, immunity and health maintenance, the composition and structure of bacterial communities within the fish intestinal ecosystem have not been extensively explored (Ni et al., 2014). For example, there are no studies describing the intestinal microbiota of *P. adspersus*. However, the microbiota of the related flatfish *P. olivaceus*, which is raised in Korea, has been examined under wild and aquaculture conditions; using a culture-dependent approach they found that *Proteobacteria* were dominant in both conditions (Kim and Kim, 2013).

The development of culture-independent methods is providing new ways to study microbial communities in their natural environments, overcoming the limitations of the culture-dependent approach. For example, high-throughput sequencing technologies have been very efficient tools for providing a more complete view of the microbial communities inhabiting organisms reared in aquaculture systems (Star et al., 2013; Trabal et al., 2014; Ghanbari et al., 2015) and have been pivotal in facilitating the discovery of gut microbiota biodiversity (Rothberg et al., 2011). However, few comparative studies have evaluated the intestinal microbiota composition in farmed versus wild fishes; reports are only available for *Salmo salar* (Holben et al., 2002), *Gadus morhua* (Dhanasiri et al., 2011), and *P. olivaceus* (Kim and Kim, 2013). Colston and Jackson (2016) debate whether work on caged animals can be used to predict the gut microbiomes of wild animals. Hence, it has been argued that studies focused on the characterization of gastrointestinal microbiota communities in hosts belonging to a natural environment are necessary (Amato, 2013). The present study compared the intestinal microbiota of *P. adspersus* obtained from different origins, wild and aquaculture, using NGS to obtain a high-resolution map of the microbiota, allowing detection of significant differences in the microbiota composition and also in the predicted functions of these microbes, which may have consequences for health and nutrition. In our study, we used an NGS approach, obtaining the first data on the intestinal microbiome of this species.

## Materials and Methods

### Sample Collection

Intestinal content samples were collected from aquaculture and wild *P. adspersus* and were immediately stored at -20°C. Fine flounder specimens from an aquaculture facility were collected from the Centro de Desarrollo Tecnológico Tongoy de Fundación Chile (CDTT) (Tongoy, Coquimbo; latitude 30.251° S, longitude 71.502° W; Chile). Five individuals (each ≈600 g) without deformities or apparent illnesses were used for feces collection. The intestinal contents were obtained by abdominal massage of fish previously anesthetized with Here-S (Bayer^®^). Fine flounder specimens from the wild environment were collected from latitude 30.104° S, longitude 71.377° W to latitude 30.302° S, longitude 70.608° W, in an area roughly surrounding the aquaculture facility. Five individuals of ≈800 g were included. Fish were caught by angling and kept in ice until processing. The intestinal contents were obtained as described by Kim et al. (2007). Briefly, the digestive tract was aseptically separated from the abdominal cavity with a scalpel, and the hindguts were squeezed to remove and collect the intestinal contents. This study was conducted in accordance with the recommendations of Guide for the Care and Use of Laboratory Animals of the National Institutes of Health, and the Committee on the Ethics of Animal Experiments of the INTA Universidad de Chile approved the protocol.

### DNA Extraction and Sequencing

DNA was extracted from the intestinal content samples (0.25 g) using the MO BIO PowerFecal^®^DNA Isolation Kit (MO BIO Laboratories, Carlsbad, CA, USA) according to the manufacturer’s protocol, including prior treatment with lysozyme (SIGMA) at 0.8 mg mL^-1^ at 37°C for 60 min and then with proteinase K (Invitrogen) 0.1 mg mL^-1^ at 37°C for 60 min. The DNA concentration was determined using the Qubit^®^ dsDNA BR Assay Kit (Life Technologies, Grand Island, NY, USA). We amplified a standard V3 region using the primers 341F and 518R (Muyzer et al., 1993) and incorporating adapters and barcodes suggested by Whiteley et al. (2012) (Supplementary Table S1). Each 30 μL PCR reaction contained 50 ng of DNA, each primer at 0.25 pM, 0.5 mM dNTPs, 1.5 mM MgCl_2_ and two units of DyNAzyme Ext DNA polymerase (Thermo). The PCR conditions were 95°C for 2 min; followed by 30 cycles of 95°C for 20 s, 50°C for 30 s, and 72°C for 5 min; followed by a final extension at 72°C for 10 min. DNA sequencing was performed by OMICS (OMICS Solutions, Santiago, Chile) via the Ion Torrent Life Technologies/314 Chip instrument platform.

### Sequence Analysis

Sequencing reads of 16S rRNA gene were processed using UPARSE (Edgar, 2013) and analyzed using QIIME (Caporaso et al., 2010). The quality of the reads was assessed using FastQC Software, and the reads were then filtered by quality and length by using the USEARCH algorithm. The resulting quality-filtered FASTA files were merged. Then, the sequences were trimmed to 140 bp, dereplicated, and sorted by abundance, and singletons were discarded. The reads were clustered into operational taxonomic units (OTUs) based on 97% identity using UPARSE with the “cluster_otu” command in USEARCH. Then, chimeric sequences were removed using the UCHIME algorithm. Taxonomic information was provided for each OTU with the “assign_taxonomy.py” QIIME script using the Ribosomal Database Project (RDP) as the reference database, with a confidence of 0.8. Representative sequences for each OTU were determined based on sequence frequency, and representative sequences were aligned using PyNAST algorithms. Phylogenetic relationships were determined based on representative sequence alignment using FastTree. Taxonomic assignments for each representative sequence were determined, and the above information was combined to construct the BIOM file using the “make_otu_table.py” QIIME script. These data are summarized in Supplementary Table S5. The graphics for the relative abundance of the composition of intestinal microbiota were performed in the R environment using the ggplot package.

### Diversity Indexes and Statistical Analyses

Good’s coverage and alpha diversity indexes, including community diversity (Simpson and Shannon index), richness (Chao-1) and phylogeny-based metrics (PD Whole Tree), were calculated using the “alpha_diversity.py” QIIME script. The Mann–Whitney test was used to test the differences in alpha diversity (Shannon Diversity index, Simpson index, richness and PD Whole Tree) between the wild and aquaculture flounder using GraphPad Prism 6 (GraphPad Software, Inc., La Jolla, CA, USA). A *p*-value < 0.05 was considered statistically significant. Beta diversity measurements were investigated through a principal coordinate analysis (PCoA) performed on the phylogenetic beta-diversity matrix, obtained by weighted UniFrac analysis, using “beta_diversity.py” QIIME script. EMPeror was used to visualize the PCoA plots from weighted UniFrac metrics.

Differential abundance of the bacterial components between wild and aquaculture flounder was assessed using linear discriminant analysis effect size (LEfSe), which considers both statistical significance and biological relevance (Segata et al., 2011). LEfSe combines the standard tests for statistical significance (Kruskal–Wallis test and pairwise Wilcoxon test) with linear discriminate analysis (LDA) to estimate the effect size of each differentially abundant feature. The alpha value for the factorial Kruskal–Wallis test is 0.05, and the threshold for the logarithmic LDA score for discriminative features is 2.0.

### Predicted Molecular Functions Based on 16S rRNA Data Using PICRUSt

To predict the metagenomes of each of the samples, a closed reference OTU-picking strategy based on the Greengenes database (version 13.5) was adopted, with a 97% sequence similarity threshold using the “pick_closed_reference_otus.py” QIIME script. The resulting OTU table, in biom format, was then used to generate inferred metagenomic data using PICRUSt (Langille et al., 2013) version 1.1.0 with the default parameters. The abundance values of each OTU were first normalized to their respective predicted 16S rRNA copy numbers. Predicted functional pathways were annotated using the Kyoto Encyclopedia of Genes and Genomes (KEGG) database. The accuracy of the predictions of the metagenomes was assessed by computing the NSTI (Nearest Sequenced Taxon Index), which is an index that indicates the relationship of the microbes in a particular sample to the bacterial genomes in a database (Supplementary Table S2). The associated metabolic pathways were identified by employing HUMAnN2 (The HMP Unified Metabolic Analysis Network) with the default settings. The *t*-test was used to identify bacterial functional pathways that were differentially abundant in intestinal microbiota of wild flounder and aquaculture flounder. All *p*-values were corrected for an FDR of 0.05. FDR corrected *p*-values bellows 0.05 (FDR < 0.05) were considered significant (Supplementary Table S3).

### Data Deposition

Raw sequences from 16S rRNA gene profiling are accessible through the following SRA study accession numbers: SRX2200382, SRX2200381, SRX2200380, SRX2200379, SRX2200378, SRX2200377, SRX2200376, SRX2200375, SRX2200374, and SRX2200373.

## Results

### Sequencing Depth

**FIGURE 1:**
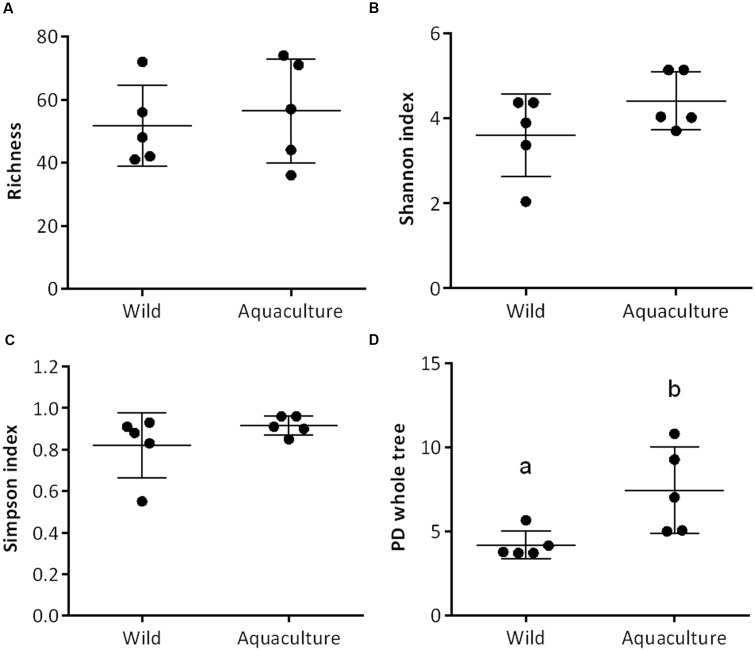
**Comparison of alpha diversity indexes between wild and aquaculture flounder (*Paralichthys adspersus*)**. Diversity in the gut bacterial community was measured using Chao-1 **(A)**, Shannon index **(B)**, Simpson index **(C)**, and phylogeny-based metrics **(D)**. Different lowercase letters above the boxplots indicate significant differences in alpha diversity between wild and aquaculture flounder (*p* < 0.05, Mann–Whitney test).

Intestinal contents were collected from *P. adspersus* (flounder), both wild (*n* = 5) and aquaculture (*n* = 5). Microbiota composition was analyzed using barcoded sequencing of the V3 region of the 16S rRNA gene. The sequences were processed and analyzed using two pipelines, UPARSE and QIIME (Caporaso et al., 2010; Edgar, 2013). After removing low-quality reads and chimeras (UPARSE), there were 71,405 remaining reads; 26,569 from wild flounder and 44,836 from aquaculture flounder. These sequences, each 140 bp in length, were assigned to 356 OTUs based on 97% similarity using QIIME. For each sample, we determined the expected richness (Chao1 index, **Figure 1**).

### Alpha and Beta Diversity

Bacterial community diversity was measured using the Shannon index, the Simpson index and a phylogeny-based metric (Phylogenetic Diversity Whole Tree, PD) as implemented in QIIME. The richness did not differ significantly between aquaculture and wild flounder; similarly, the diversity indexes, both Shannon and Simpson, showed no significant differences. In contrast, the phylogeny-based metric, PD, was significantly higher for aquaculture flounder than for wild flounder according to the non-parametric Mann–Whitney test, *p* < 0.05 (**Figure 1**).

**FIGURE 2:**
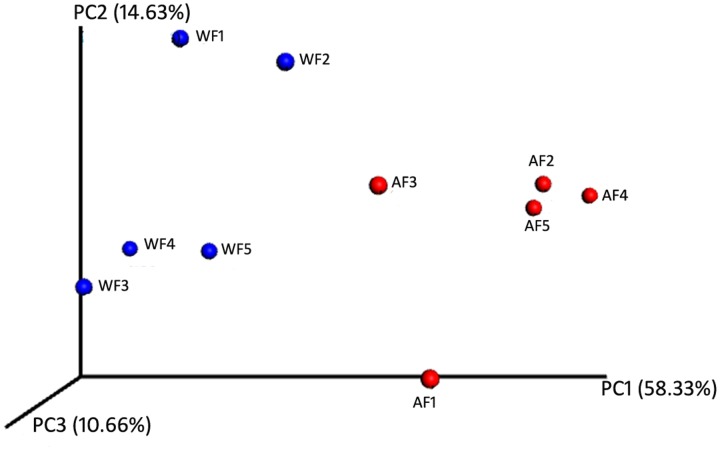
**Similarity among the bacterial communities associated with *Paralichthys adspersus***. Principal coordinate analysis (PCoA) based on weighted UniFrac analysis of bacterial profiles. Circles represent individual samples from *P. adspersus* intestinal microbiota. Red circles correspond to samples derived from aquaculture, and blue circles correspond to samples from wild fish.

The beta-diversity of the bacterial communities associated with flounder in the two conditions, wild and aquaculture, was investigated through a PCoA performed on the phylogenetic beta-diversity matrix obtained by UniFrac (**Figure 2**). The first two components explain a total of 72.96% of the variation (first component, 58.33%; second component, 14.63%).

### Dominant Components of the Microbiota in Wild and Aquaculture *P. adspersus*

**FIGURE 3:**
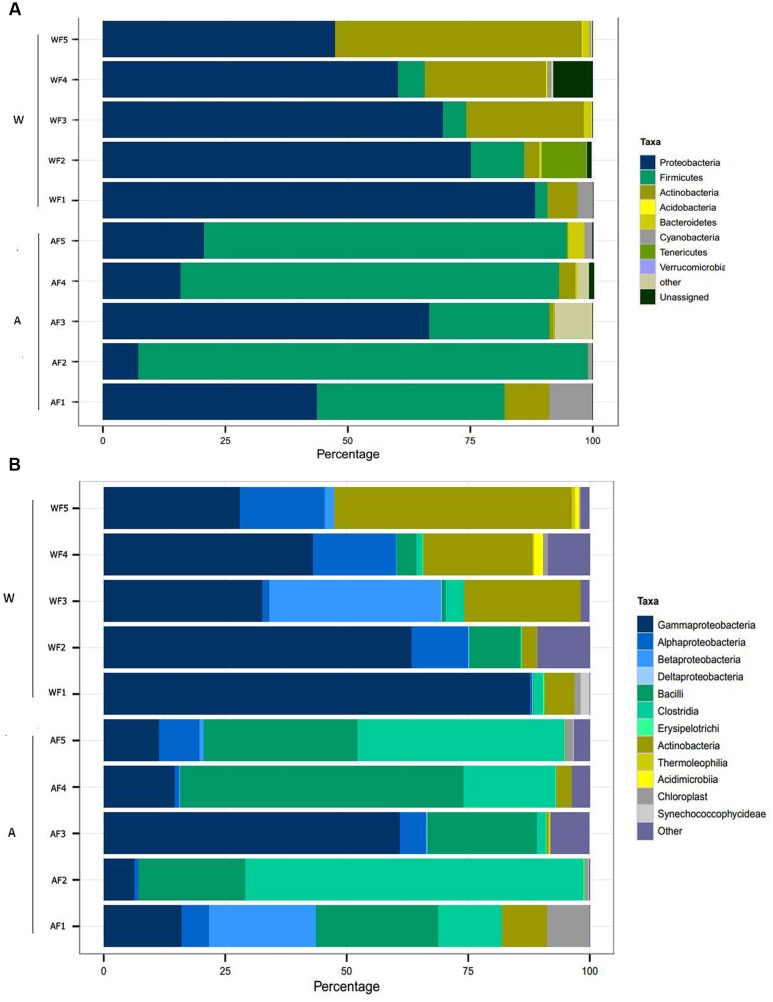
**Relative abundance of OTUs at the phylum (A)** and class **(B)** levels in the intestinal microbiota from wild and aquaculture *P*. *adspersus*. In the figure, W corresponds to individual wild fish (Wild flounder, WF1 to WF5) and A corresponds to aquaculture fish (Aquaculture flounder, AF1 to AF5). The relative abundance was calculated based on taxonomy assignment using the RDP database.

The results of the taxonomic assignment analysis at the phylum and class levels are shown in **Figure 3**. The most abundant and common phyla in both origin conditions (aquaculture and wild) were *Proteobacteria*, *Firmicutes* and *Actinobacteria*, which were present in at least 90% of the samples. In terms of common classes, *Gammaproteobacteria*, *Alphaproteobacteria*, *Bacilli*, *Clostridia*, and *Actinobacteria* were the most abundant and were present in at least 80% of the samples. Recent reports have defined the core microbiome as those components present in >80% of individuals examined (Lokesh and Kiron, 2016); according to this criterion, these classes could be considered the core microbiome of fine flounder.

**FIGURE 4:**
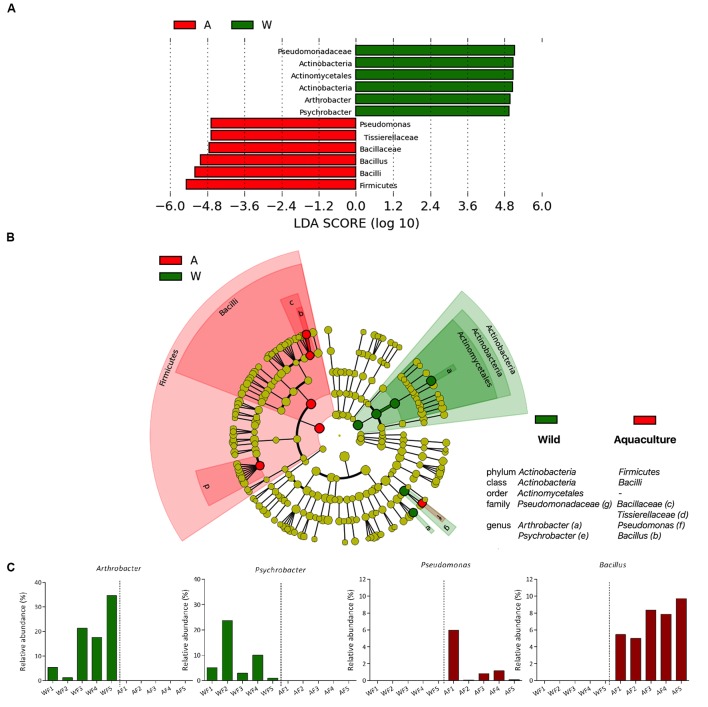
**Differences in the microbiota between wild and aquaculture flounder**. Analysis of 16S rRNA reveals the differential composition of microbiota depending on the origin of the sample. LEfSe was used to determine the statistical significance and the effect size of the differential abundance of taxa between wild and aquaculture flounder. **(A)** LDA score of abundance of taxa; **(B)** cladogram showing differentially abundant taxa (phylum to genus) of the intestinal microbiota of flounder under two conditions; **(C)** relative abundance of bacterial genera that differ significantly between wild and aquaculture flounder.

However, the intestinal microbiota of flounder under conditions of aquaculture presented differences in composition compared to wild flounder. The most abundant phylum in wild flounder was *Proteobacteria*, averaging 68.1 ± 15.4% in relative abundance; in contrast, in aquaculture flounder, this phylum averaged 30 ± 24% relative abundance. Reciprocally, the most abundant phylum in aquaculture flounder was *Firmicutes*, averaging 61.2 ± 28.4%; in contrast, this phylum shows low abundance in wild flounder averaging 4.7 ± 4% (**Figure 3A**). This difference is statistically significant according to LEfSe (see below). The phylum *Actinobacteria* shows more abundance in wild flounder, ranging from 3.2 to 50.4%, while in aquaculture fish it averages 0.7 ± 1.3%, and this difference is statistically significant (**Figure 4A**). Other phyla such as *Acidobacteria, Bacteroidetes, Cyanobacteria, Tenericutes*, and *Verrucomicrobia* showed low abundance in both conditions (**Figure 3A**).

At the next taxonomic level, the most abundant class within *Proteobacteria* was *Gammaproteobacteria* (**Figure 3B**). In wild flounder, this class showed 30–87% relative abundance, whereas in aquaculture fish it ranged from 6 to 61%. *Bacilli* and *Clostridia* were the most abundant class within the *Firmicutes* phylum. In aquaculture flounder, *Bacilli* showed 22–58% relative abundance and *Clostridia* ranged from 2 to 69%. Wild flounder showed contrasting results for these classes, which only reached relative abundances of 10 and 3%, respectively. Furthermore, LEfSe analysis indicates that *Bacilli* corresponded to the class with a significant difference between wild and aquaculture flounder (**Figures 4A,B**). In wild flounder, *Actinobacteria* showed 3–48% relative abundance; in contrast, this phylum showed low relative abundance in aquaculture flounder (1–9%). This difference was statistically significant according to LEfSe analysis (**Figures 4A,B**).

### Bacterial Populations Significantly Associated with Origin (Wild or Aquaculture)

Specific taxa that were differentially distributed between wild and aquaculture flounder were identified using LEfSe. This approach allows significant differences in the relative abundance of each taxon to be identified based on statistical tools. The results are shown in **Figure 4**, which depicts each phylum, class, order, family and genus presenting a significant difference between the two conditions, aquaculture (red) and wild (green). **Figure 4A** shows these data as a bar plot of the LDA score for both conditions; **Figure 4B** shows these differences in a cladogram. The phyla showing significant differences between the two conditions are *Firmicutes* and *Actinobacteria*, which were detected in aquaculture and wild flounder, respectively. At the genus level, a total of four genera were differentially represented between the two conditions. *Bacillus* and *Pseudomonas* were represented in aquaculture flounder, whereas *Arthrobacter* and *Psychrobacter* were observed in wild flounder (**Figure 4C**).

### Functional Pathways Showing Significant Differences

**FIGURE 5:**
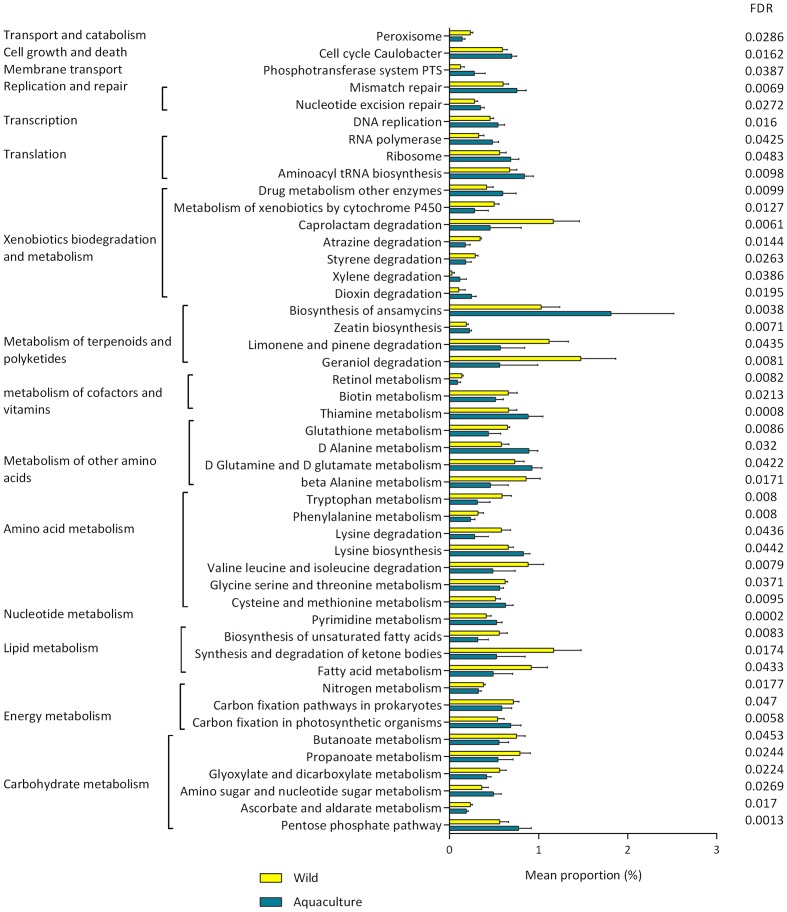
**Presumptive functions of the intestinal microbiota from wild and aquaculture *P. adspersus* based on differential abundance**. PICRUSt was used to predict the metagenome, and HUMAnN2 was used to find the associated metabolic pathways. The pathways showing significantly different abundance were identified using the *t*-test (*p*-value < 0.05). All *p*-values were corrected for an FDR of 0.05. FDR corrected *p*-values below 0.05 (FDR < 0.05) were considered significant.

The changes in the presumptive functions of the intestinal microbiota of *P. adspersus* were examined by predicting the metagenomes using PICRUSt. The accuracy of the prediction was evaluated by computing the NSTI, and the mean of the samples was 0.091 ± 0.029. Twenty-six functional pathways were found to be more highly abundant in wild flounder, including pathways related to lipid metabolism, biodegradation of xenobiotics, and metabolism of terpenoids and polyketides. In the case of aquaculture flounder, 22 pathways were found to be more highly abundant with respect to wild flounder, including those related to amino acid metabolism, carbohydrate metabolism, and nucleotide metabolism. This information is detailed in **Figure 5**.

## Discussion

*Paralichthys* includes 17 species, which are distributed along the coasts of Atlantic, Indian and Pacific Oceans. In Chile, many species belonging to this genus are registered, with *P. adspersus* being the most important for its wide distribution and predominance (Bahamonde and Pequeño, 1975); nevertheless, to date, no studies have described the intestinal microbiota of this fish. Previously, Kim and Kim (2013) evaluated the microbiota associated with the intestinal mucosa of *P. olivaceus* from both wild and farmed populations. These authors characterized the microbiota using a culture-dependent approach including 100 bacterial isolates, 51 from farms and 57 of wild origin. In both cases, the most abundant phylum was *Proteobacteria*, with an average richness of 55%, followed by *Firmicutes* and *Bacteroidetes* with 30 and 11%, respectively. Interestingly, these authors report that the only significant difference between wild and farmed fishes corresponded to the phylum *Firmicutes*, at 42 and 18.4%, respectively. Our results contrast with those described by Kim and Kim (2013) because we show two phyla associated with different origins; *Firmicutes* was significantly associated with farmed fish, and *Actinobacteria* was significantly more prevalent in wild fishes.

Our main observation is the significant difference in the microbiota depending on the origin of the fish (wild or farmed), as well as the predicted functions of those differentially distributed bacteria. When analyzing beta diversity, we observed that microbial communities were differentially grouped according to their origin (**Figure 2**). Measures of beta diversity can elucidate how much diversity is unique to a local assemblage or to ecological processes, such as habitat filtering or competition (Lozupone and Knight, 2008). The artificial environment in farming systems, in which various parameters such as water quality, diet, population density and habitat are different from those in natural environments, may lead to the establishment of a different intestinal microbiota compared to that in wild fishes of the same species (Strøm and Olafsen, 1990; Martin-Antonio et al., 2007; Kim and Kim, 2013). Recently, Smith et al. (2015) reported that the microbiota of three-spine stickleback sampled in different locations showed different compositions. Their results indicate that these among-location differences might be associated with variables such as habitat type and food-associated microbiota. On the other hand, other recent reports showed that the diversity of the intestinal bacterial community declines in response to artificial feeding. This is the case for Atlantic cod (*Gadus morhua*) and sea bream (*Sparus aurata*) as reported by Dhanasiri et al. (2011) and Kormas et al. (2014), respectively. This is very interesting because our results indicated that diversity was similar between aquaculture flounder and wild flounder, except for the PD whole tree, which showed a significant difference.

Recent reviews addressing the teleost microbiome highlight the presence of representative phyla such as *Proteobacteria*, *Firmicutes*, *Actinobacteria*, *Fusobacteria*, and *Bacter*o*idetes* in various species of fish (Llewellyn et al., 2014; Colston and Jackson, 2016). The review by Llewellyn et al. (2014) does not include fishes from the genus *Paralichthys*, instead evaluating the flatfish *Solea senegalensis* from a farming origin, in which the main phyla were *Proteobacteria* and *Firmicutes* (Martin-Antonio et al., 2007). In the present study, *Proteobacteria* showed the highest relative abundance (68 ± 15%) in wild flounder; in contrast, this phylum in aquaculture flounder showed only 30 ± 24% relative abundance. Although *Proteobacteria* does not present a significant difference between the two origins, members of this phylum are differentially distributed at the genus level. For example, the genus *Psychrobacter* is significantly more abundant in wild flounder than in aquaculture flounder. The genus *Psychrobacter*, which belongs to the family Moraxellaceae within the *Gammaproteobacteria*, has been reported to be dominant in the gut of fast-growing grouper (Sun et al., 2009), and these bacteria can inhibit pathogenic *Vibrio*. In Atlantic cod, *Psychrobacter* isolates showed enzymatic activities such as protease, chitinase and phytase, all of which are useful for nutrition (Askarian et al., 2013). In addition, the genus *Pseudomonas* is significantly more abundant in aquaculture flounder than in wild flounder. *Pseudomonas* is a diverse bacterial group showing a wide variety of metabolic abilities, broad ecological distribution and adaptability to a range of environmental niches. *Pseudomonas* strains have been used as probiotics in aquaculture, improving the response to pathogens in different hosts (Alavandi et al., 2004; Giri et al., 2014).

The phylum *Actinobacteria* was more abundant in wild flounder, with *Arthrobacter* as the major representative genus. *Arthrobacter* spp. correspond to gram-positive bacteria belonging to the class *Actinobacteria*. This genus is relatively common on the aerial surface of plants and has been reported in marine and freshwater fish and other seafood. Bacterial isolates belonging to this genus have been reported as producers of antimicrobial compounds (Hentschel et al., 2001; Jayanth et al., 2001) and valuable substances such as amino acids, vitamins, enzymes, specific growth factors, pigments and polysaccharides (Abrashev et al., 1998), conferring advantages for use as probiotics in aquaculture, especially in shrimp (Li et al., 2006, 2008; Xia et al., 2014).

The phylum *Firmicutes* was significantly more abundant in aquaculture flounder, with *Bacillus* as the major representative genus. *Bacillus* species are gram-positive, spore-forming bacteria, used commercially as probiotics (Wang et al., 2008). They have been described as beneficial in the farming of aquatic organisms due to increases in survival and growth. Aly et al. (2008) suggest *Bacillus subtilis* as a potential probiotic for growth in *Oreochromis niloticus* due to its antimicrobial activity against bacterial pathogens. Cha et al. (2013) evaluated the dietary supplementation of *Bacillus* strains in olive flounder and its response to infection with *Streptococcus iniae.* Fish fed *Bacillus* showed significantly higher survival rates.

Assessing which bacterial communities are present in a greater proportion in each condition is important because it provides information on the relationships of these communities with their host and in turn on the contributions of these communities. This will allow the greater abundance of certain bacterial genera to be associated with the functionality that those bacteria could provide within the host. The changes in the presumptive functions of the intestinal microbiota community of *P. adspersus* were examined by predicting the metagenomes using PICRUSt. The accuracy of metagenome predictions depends on how closely related the microbes in a given sample are to microbes with sequenced genome representatives, as measured by the NSTI, with lower values indicating a closer mean relationship (Langille et al., 2013). The NSTI values for the intestinal microbiota samples of *P. adspersus*, with the mean of the samples being 0.091 ± 0.029, are considered a good data set for examining predictions from PICRUSt, given that they are in the range proposed by Langille et al. (2013), 0.03 ± 0.2 for human samples and 0.17 ± 0.02 for more diverse communities such as soil samples.

In wild flounder, 26 functional pathways were found to be differentially abundant (**Figure 5**), including those related to the lipid and carbohydrate metabolism, biodegradation of xenobiotics, metabolism of terpenoids and polyketides, and metabolism of cofactors and vitamins. The metabolism of biotin is presented as a significant pathway in wild flounder; it has been reported that an important source of biotin supply to animals is the endogenous production by intestinal microbiota (McMahon, 2002). Regarding the biodegradation of xenobiotics, the pathways that are significantly increased are the degradation of caprolactam, atrazine, and styrene. Caprolactam, a precursor for the synthesis of polyamide fibers, can exert toxic effects on various lifeforms (Shama and Wase, 1981). Polyketides have displayed a wide array of bioactive properties, with beneficial effects for the host, such as antibacterial and antiviral activities (Fan et al., 2017). Furthermore, a diverse range of other classes of compounds with interesting activities occur across symbiotic associations and habitats, including organic acids, phenolics and terpenes (Flórez et al., 2015). Regarding carbohydrate and lipid metabolism, short-chain fatty acids (SCFAs), mainly propionate and butyrate, were abundant in wild flounder; these SCFAs are the main energy source for colonic epithelial cells and have profound effects on gut health (Maslowski and Mackay, 2011). Additionally, it has been proposed that SCFAs stimulate the immune system (Llewellyn et al., 2014). Another important pathway in wild fish was the biosynthesis of unsaturated fatty acid, including essential omega-3 fatty acids such as eicosapentaenoic acid (EPA) and docosahexaenoic acid (DHA). Many of the previously reported EPA/DHA bacterial producers are members of the *Gammaproteobacteria* (Dailey et al., 2016), which is dominant in wild flounder (**Figure 3**).

For aquaculture flounder, 22 pathways were found to be differentially abundant with respect to wild flounder, including pathways related to the metabolism of terpenoids and polyketides, amino acid metabolism, and carbohydrate metabolism. Within the metabolism of terpenoids and polyketides, the biosynthesis of ansamycins is significantly greater in aquaculture flounder. Some examples of ansamycins are the antibiotic rifamycin and the anti-tumor compounds geldanamycin and ansamitocin P-3 (Rude and Khosla, 2006). This pathway may be used as protection against bacterial pathogens in aquaculture fish in the context of the high density of organisms in intensive fish farming. Pathways related to carbohydrate metabolism are increased in aquaculture flounder, including the pentose phosphate pathway and the metabolism of amino sugar and nucleotide sugar. This might be related to the artificial diet (Supplementary Table S4), which contains 11% carbohydrates. This diet strongly contrasts with that of wild fish; *P. adspersus* has been described as a carnivorous fish, and its most frequent preys are anchovy (*Engraulis ringens*) and crustaceans. Some reports from northern Chile indicate that anchovy can be observed in the contents of more than 90% of the guts (Silva and Oliva, 2010). The nutritional composition of anchovy meal in Chile is 73.8% crude protein and 8.4% crude fat (Lee et al., 2002), revealing important differences in the feeding between wild and reared flounder. As has been observed in many animal systems, this factor might influence the microbiota composition and their metabolic functions (Montagne et al., 2003; Bakke-McKellep et al., 2007).

Our results highlight the difference in the microbiota composition between wild and reared fish from the phylum level to the genus level. This is very important in the context of co-evolution between the host and its microbiota because co-evolution is believed to have been an important mechanism in the formation of the host–gut microbe relationship. However, factors such as diet may influence the composition of the gut microbiota and consequently the metabolic functions of intestinal microbes, with effects on the host’s nutrition and immune defense. In summary, this is the first in-depth characterization of the microbiota in the fine flounder *P. adspersus*, and this analysis reveals substantial differences between wild and reared specimens, including disparities at the phylum and class levels. Furthermore, in both cases, predicted functions (metabolic pathways) indicated that those microbiota might provide beneficial effects for the host, but wild flounder showed more noteworthy pathways (EPA/DHA, SCFA, biotin). An interesting projection of these results will be to obtain bacterial isolates from *P. adspersus* and evaluate their beneficial effects on the host, such as increasing the nutritional value or improving the immune response, as recently reported by Beck et al. (2016) in the case of olive flounder.

## Author Contributions

CR: data collection; data analysis and interpretation; drafting the article. JR: conception or design of the work; sample collection; writing and critical revision of the article; final approval of the version submitted.

## Conflict of Interest Statement

The authors declare that the research was conducted in the absence of any commercial or financial relationships that could be construed as a potential conflict of interest.
